# Urological myriad

**DOI:** 10.1590/S1677-5538.IBJU.2018.03.01

**Published:** 2018

**Affiliations:** 1 Divisão de Urologia do A.C. Camargo Cancer Center Fundação A. Prudente, São Paulo, Brasil

The internationality and diversity of subjects have been remarkable characteristics of Internatio-nal Braz J Urol, an open free access urological journal, as is this issue.

Collaborative multicenter studies in Urology are lacking in Latin American; efforts in this way are welcome by International Braz J Urol. Colleagues from Mexican centers and São Paulo University (Manzo and Vicentini, respectively), found differences in the management of kidney stones with percutaneous nephrolitotomy when compared trained and non-trained urologists. In that survey, they evaluated data from 331 urologists from 15 Latin American countries, that answered a 27-item questionnaire, easily accessed by mobile phones. Patel and Monga, from Cleveland Clinic, present in this issue, the distinct physical and mechanical properties of different ureteral sheaths, that are commercially used in their country, reiterating the possible impact in clinical practice.

Every year, thousands of men worldwide develop recurrence of prostate cancer after the use of external beam or interstitial therapy for primary tumor treatment. Although the vast majority of these patients has been treated with androgen deprivation therapy, local salvage treatments can offer oncological control without the metabolic side effects of hormonal therapy. However, local salvage procedures may result in several urinary and sexual alterations. Recently, the literature is profuse in debates regarding the pros and cons of several options for second local treatment in this population. In the difference of opinion section, we have confronts of arguments favoring salvage radical prostatectomy (Drs. Reis from UNICAMP, Campinas, São Paulo, Brazil and Nguyen from Dana Faber, from Boston, USA), salvage cryotherapy (from Denver University group, with Drs. da Silva and Kim), and about salvage HIFU (A.C. Camargo's' team, from São Paulo).

Active surveillance is a changing paradigm in the last years for stage I testis cancer. Dr. Jain et al. after a national survey that encompassed more than 300 radiation oncologists from United states, observed that a significant percentage of them are not confident in offering surveillance for stage I seminoma patients; there are concerns about the real adherence of patients to the protocols and fears of results of salvage treatments in cases of progression, among other reasons.

Contrary to verified in Eastern world, a Shanghai's group revealed in a large cohort of Eastern Chinese descendants, that the polymorphisms of PCA3 marker were not associated with prostate cancer risks, warranting the need for more investigations about cancer heterogeneity in different ethnicities on Earth.

Moving to uropediatry, Turkish colleagues from Hasekiand Istanbul, demonstrated almost 70% of success with endoscopic use of a non-biodegradable compound in 39 children with vesicoureteral reflux. Uropediatric team from Tehran University, describe the positive results of the use of a plastic wrap barrier as a coverage for the exposed mucosal template in bladder extrophy, before surgical reconstruction. Reduction of polyps size, skin allergy reactions, without pre-malignant alterations were described with this polyvinylidene chloride (PVdC) barrier.

Three groups from Rio de Janeiro (Hospitals Souza Aguiar, da Lagoa and State University of Rio de Janeiro, UERJ) described the characteristics of penile fracture in a group of men who have sex with men (MSM). They found a specific sexual position associated with these severe fractures and they reported that in all cases diverse sexual dysfunctions (erectile dysfunction, orgasmic and ejaculatory abnormalities, pain during intercourse) or aesthetical dissatisfaction were present after the treatment in all cases of MSM group. Researchers from Italy (Trieste and Bologna) and from London, investigated the differences of patients' perception of their penile angulation in comparison with objective measures performed by experts. There were no conformities in perceptions of the curvatures in patients with Peronye's Disease in men with congenital penile curvature.

Lourenço et al. from Hospital Albert Einstein and Santa Casa de São Paulo, reported similarity in the functional outcomes and quality of life with the use of homemade and commercial transobturatory slings, for female urinary incontinence. This finding is relevant in developing countries, where healthy costs are a permanent question when synthetic materials are used in surgery.

History of Urology in an interesting field of knowledge. Antonio Marte from Bologna, presents a huge historical manuscript focused in varicocele, containing interesting information and illustration from ancient times until nowadays.

In the video section, we have interesting movies from several continents. Among these videos, that of Dr. Almeida e Paula et al., from Presidente Prudente, Brazil, demonstrated a challenging case: a laparoscopic resection of a 10 cm right side functional adrenal pheocromocytoma in a 24-week pregnant woman, with prominent symptoms and inferior vena cava compression.

Enjoy yourself with the myriad of international Urological experiences in this International Braz J Urol edition.

